# Fractional-Dose Inactivated Poliovirus Vaccine Campaign — Sindh Province, Pakistan, 2016

**DOI:** 10.15585/mmwr.mm6647a4

**Published:** 2017-12-01

**Authors:** Aslam Pervaiz, Chukwuma Mbaeyi, Mirza Amir Baig, Ashley Burman, Jamal A. Ahmed, Sharifa Akter, Fayaz A. Jatoi, Abdirahman Mahamud, Rana Jawad Asghar, Naila Azam, Muhammad Nadeem Shah, Mumtaz Ali Laghari, Kamaluddin Soomro, Mufti Zubair Wadood, Derek Ehrhardt, Rana M. Safdar, Noha Farag

**Affiliations:** ^1^National Stop Transmission of Polio (N-STOP) Program, Field Epidemiology and Laboratory Training Program, Pakistan; ^2^Global Immunization Division, Center for Global Health, CDC; ^3^Polio Eradication Department, World Health Organization, Geneva, Switzerland; ^4^National Emergency Operations Centre for Polio Eradication, Islamabad, Pakistan; ^5^Sindh Province Emergency Operations Centre for Polio Eradication, Pakistan; ^6^Resident Advisor, Field Epidemiology and Laboratory Training Program, Pakistan; ^7^Armed Forces Postgraduate Medical Institute, Rawalpindi, Pakistan.

Following the declaration of eradication of wild poliovirus (WPV) type 2 in September 2015, trivalent oral poliovirus vaccine (tOPV) was withdrawn globally to reduce the risk for type 2 vaccine-derived poliovirus (VDPV2) transmission; all countries implemented a synchronized switch to bivalent OPV (type 1 and 3) in April 2016 (*1*,*2*). Any isolation of VDPV2 after the switch is to be treated as a potential public health emergency and might indicate the need for supplementary immunization activities (*3*,*4*). On August 9, 2016, VDPV2 was isolated from a sewage sample taken from an environmental surveillance site in Hyderabad, Sindh province, Pakistan. Possible vaccination activities in response to VDPV2 isolation include the use of injectable inactivated polio vaccine (IPV), which poses no risk for vaccine-derived poliovirus transmission. Fractional-dose, intradermal IPV (fIPV), one fifth of the standard intramuscular dose, has been developed to more efficiently manage limited IPV supplies. fIPV has been shown in some studies to be noninferior to full-dose IPV (*5*,*6*) and was used successfully in response to a similar detection of a single VDPV2 isolate from sewage in India (*7*). Injectable fIPV was used for response activities in Hyderabad and three neighboring districts. This report describes the findings of an assessment of preparatory activities and subsequent implementation of the fIPV campaign. Despite achieving high coverage (>80%), several operational challenges were noted. The lessons learned from this campaign could help to guide the planning and implementation of future fIPV vaccination activities.

## Campaign Preparations and Implementation

**FIGURE 1:**
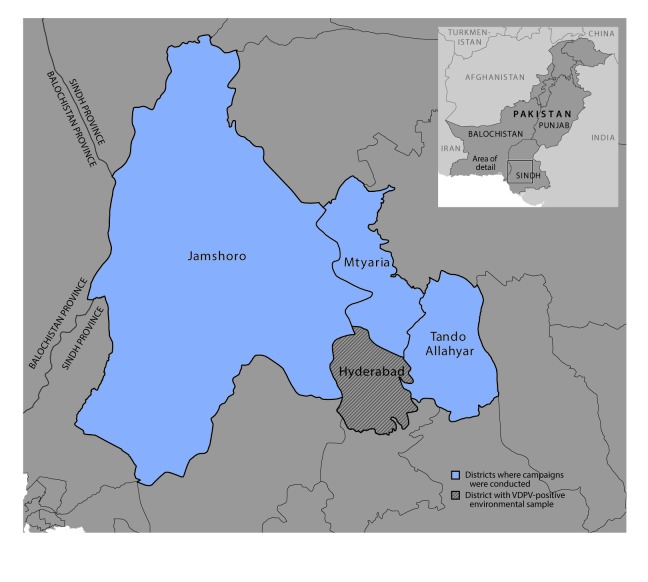
Location of fractional inactivated poliovirus vaccine campaign — Sindh Province, Pakistan, October–November 2016 **Abbreviation:** VDPV = vaccine-derived poliovirus.

The fIPV campaign was conducted in 120 subdistricts, known as union councils, in Hyderabad and three neighboring districts (Jamshoro, Matyari, and Tando Allahyar) of Sindh province during October 24–November 1, 2016 (Figure 1). Areas with sewage drainage to the Tulsidas Pumping Station, from which the VDPV isolate was identified, and those within the potential zone of poliovirus circulation were chosen for campaign implementation. The target population for the campaign comprised 258,492 children aged 4–23 months (Table). In contrast to OPV campaigns, in which house-to-house visits constitute the primary strategy for vaccination activities, the fIPV campaign was conducted at fixed sites, such as hospitals and dispensaries, and through deployment of outreach teams to designated vaccination stations. This was to ensure cold chain maintenance and safe injection practices.

**TABLE:** Distribution of union councils, target populations, and vaccination sites during a fractional inactivated poliovirus vaccine campaign, by district — Sindh Province, Pakistan, October–November 2016

District	No. of union councils	Target population	Sites visited by campaign assessors	
			No. of fixed sites	No. of outreach stations
Hyderabad	54	99,392	54	211
Jamshoro	28	62,376	33	143
Matyari	18	48,524	22	120
Tando Allah Yar	20	48,200	27	116
**Total**	**120**	**258,492**	**136**	**590**

Vaccinators for the fIPV campaign were recruited mostly from among Pakistan’s Lady Health Worker Programme* and staff members of the national Expanded Programme on Immunization (EPI). A small proportion of vaccinators were recruited from among other community health workers, including dispensary staff members, clinical ward attendants, and, in one district, gardeners who had previous experience working in EPI. In addition to vaccinators, team assistants and social mobilizers were also recruited. A total of 995 vaccinators, 995 team assistants, and 1990 social mobilizers were recruited to support the campaign.

Vaccinators received a 2-day training and worked with teams of supervisors to develop microplans ahead of the campaigns. These microplans included details of the specific number of children within the target age group for each location as well as management of vaccine and cold chain supplies. Social mobilizers were recruited to promote awareness of the campaigns in the union councils where they were scheduled to take place. In addition, awareness of the campaign was promoted using posters and banners, radio and television, public announcements, and through engagement of religious and community leaders.

Over 9 days of the campaign, vaccination activities took place at designated locations during 8 a.m.–4 p.m. A team comprising at least one vaccinator, an assistant, and two social mobilizers staffed each vaccination site. Vaccinators administered a 0.1-mL dose of fIPV (one fifth of a full intramuscular dose of IPV, drawn from a vial containing 10 full intramuscular doses) intradermally in the upper left arm of each child, and assistants marked the left fifth finger of each vaccinated child with an indelible marker and entered records in a tally sheet.

## Intracampaign Monitoring and Field Assessment

A team of 20 campaign assessors drawn from the Pakistan Field Epidemiology and Laboratory Training Program monitored the campaign in 21 union councils. The 21 union councils were randomly selected from the pool of 120 union councils that took part in the campaign, with probability of selection proportional to estimated size. Campaign assessors visited selected vaccination sites, fixed and outreach, where they assessed staffing patterns and vaccine delivery procedures, including the quality of intradermal injections, vaccine supply and cold chain management, and compliance with the open-vial policy^†^ (*8*).

Among 726 (590 outreach and 136 fixed) vaccination sites visited (Table), 74 (10%) were either nonfunctional or experienced delays in commencing their activities. Of the 566 functional outreach vaccination sites, 32% were not at the locations indicated in the campaign microplan. Furthermore, vaccinators at 67 (12%) outreach stations were different from those listed in the microplan. All but one (134 of 135) of the vaccinators at fixed sites reported having previous experience with intradermal injections, compared with 90% of vaccinators at outreach stations. Nine percent of vaccinators stated that the training they received did not adequately prepare them for administering intradermal fIPV injections during the campaign.

**FIGURE 2:**
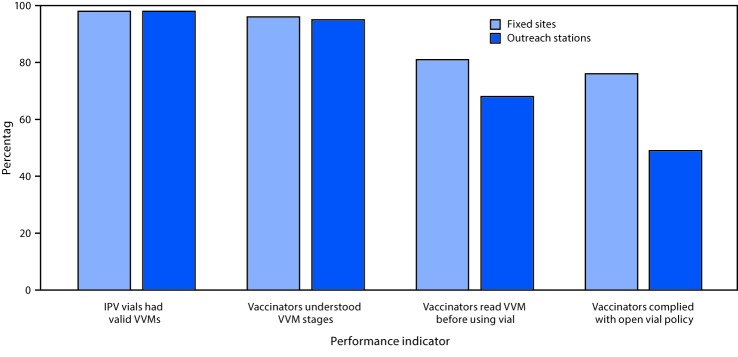
Knowledge of vaccine vial monitors and compliance with open vial policy among vaccinators during a fractional inactivated poliovirus vaccine campaign — Sindh Province, Pakistan, October–November 2016 **Abbreviations:** IPV = inactivated polio vaccine; VVM = vaccine vial monitor.

All IPV vials were within their expiration dates, and 98% had valid vaccine vial monitors (VVMs), thermochromic labels that change color when the vaccine has not been maintained at the appropriate temperature. Although 95% of vaccinators were knowledgeable about the different stages of VVMs and their significance related to vaccine viability, 32% of vaccinators at outreach stations and 19% at fixed sites were observed to not review VVMs before administering the vaccine (Figure 2). Each campaign assessor observed an average of three intradermal fIPV injections per vaccination station visited. Among 1,960 injections observed, 96% were administered at the appropriate site; bleb formation, indicative of intradermal delivery of fIPV, was observed in 82% of injections. Blebs were more commonly observed among children vaccinated at fixed sites (92%) than at outreach stations (80%). There were no adverse event reporting forms at 119 (17%) of the stations visited, and 15% of vaccinators at outreach stations were not aware of procedures for reporting adverse events. There was also considerable confusion about the open-vial policy: 51% of outreach stations and 24% of fixed sites were not reusing open IPV vials the next day, even if the VVM was valid, there was no leakage from the vial septum, and the vaccine was within its expiration date (Figure 2).

To assess the level of campaign awareness, campaign assessors interviewed 1,968 caregivers at vaccination sites. Seventy percent of caregivers were from rural union councils and 30% from urban union councils. Awareness of the campaign before its commencement was lower among caregivers from rural union councils (57%) than among those from urban union councils (83%). Of the 1,273 (65%) caregivers who were aware of the campaign, three-quarters gained their awareness through a single information source. Among this group, the principal sources of information about the campaign were social mobilizers (75%) and vaccinators/health workers (15%). Mass media, such as radio and TV, accounted for <5% of caregiver campaign awareness. Despite the pivotal role of social mobilizers in creating awareness in the community, deficiencies were noted in their performance. Among 517 social mobilizers, 63% did not have a social mobilization plan with a route map and 17% did not have a checklist to mark off houses they had visited.

## Postcampaign Coverage Assessment

A multistage cluster survey was used to assess the quality of the fIPV campaign. Thirty-five union councils were selected as primary sampling units from among 120 union councils that took part in the campaign, with probability of selection proportional to estimated size. Within each council, six neighborhood clusters were selected, from among which 10 households were randomly chosen. One eligible child in each of these households was checked for finger-marking as evidence of vaccination. Data on vaccination status based on parental recall was also collected. The postcampaign assessment took place during November 3–6, 2016 and was undertaken by staff members of the Pakistan Polio Eradication Initiative.

Overall, 2,100 children were assessed for vaccination during the campaign. Estimated coverage, accounting for the first stage clusters, was 82% (Wilson confidence interval = 78%–85%) based on finger-marking and 90% (Wilson confidence interval = 88%–92%) based on parental recall. Nearly half (49%) of 377 children reported as unvaccinated were classified as such based on the absence of finger-marking, despite claims by their parents that they were vaccinated. Among the remaining 191 children, refusals (27%), lack of awareness (24%), and absence of the child during the campaign (16%) were the main reasons for children not being vaccinated. Refusals were driven mostly by fear or illness of the child at the time of the campaign, but they only constituted approximately 2% of the 2,100 children assessed during the survey.

## Discussion

Although relatively high vaccination coverage (82%) was achieved during the fIPV campaign in response to the VDPV2 isolate in Hyderabad, the campaign highlighted several operational challenges associated with the use of an intradermally injected vaccine during a polio campaign. Many of these challenges are related to the fact that campaigns using injectable vaccines are better suited to fixed-site implementation, as opposed to the house-to-house strategy used for most polio campaigns, because of the operational complexities of safely administering injections and disposing of needles and syringes.

Many of the difficulties encountered in the fIPV campaign occurred more commonly at outreach stations. Compared with those at fixed sites, vaccinators at outreach stations were more likely to be inexperienced and to administer vaccines incorrectly. Collectively, vaccinators at both fixed sites and outreach stations could have benefited from better training and more rigorous precampaign planning. Further, all vaccinators need to be properly trained on procedures for reporting adverse events. Although social mobilizers were the principal drivers of awareness, the quality of mobilization activities before and during the campaign was suboptimal, especially in rural union councils. Detailed microplanning, including the creation and use of route maps, should be prioritized to facilitate social mobilization activities for future campaigns.

An earlier fIPV campaign in India demonstrated the operational feasibility of achieving high vaccination coverage with the vaccine in response to VDPV2 isolation from sewage (*7*). The fIPV campaign in Pakistan corroborates the feasibility of achieving high coverage, but it also highlights the operational challenges encountered during such campaigns. Current World Health Organization protocol recommends monovalent OPV type 2 (mOPV2) as the appropriate response vaccine when circulation of VDPV2 is confirmed because of its effectiveness in interrupting poliovirus transmission (*9*). IPV use is not routinely recommended, largely because it might diminish the ability to achieve high quality rounds of mOPV2 vaccination that will stop poliovirus transmission and because IPV recipients, while protected against paralysis, can continue to transmit poliovirus in an ongoing outbreak. If, however, a country elects to respond to a single VDPV isolate with fIPV, meticulous planning and preparation is required to ensure judicious and effective use of the limited global IPV stock (*1*,*10*).

**Summary**

**What is already known about this topic?**

Wild poliovirus type 2 was declared eradicated in September 2015, prompting a synchronized switch from trivalent to bivalent (types 1 and 3) oral poliovirus vaccine in April 2016. Any subsequent isolation of vaccine-derived poliovirus type 2 (VDPV2) following the switch represents a potential public health emergency for which response activities might be warranted. Vaccination options for these activities include the use of monovalent oral poliovirus vaccine type 2 (mOPV2) and/or inactivated poliovirus vaccine (IPV) for polio vaccination campaigns.

**What is added by this report?**

Because of the limited global stock of IPV, fractional-dose intradermal IPV (fIPV), which is one fifth of the full intramuscular dose, has been developed and is being used for polio vaccination activities. Several studies have indicated that fIPV is not inferior to full dose IPV, and it has been used successfully for polio response activities in India. In response to a VDPV2 isolate from sewage samples taken from Hyderabad, Pakistan, fIPV was used in a polio vaccination campaign targeting children aged 4–23 months in four districts of Sindh province, Pakistan. Although relatively high coverage (82%) was achieved, operational challenges related to the use of an intradermally injected vaccine were encountered during the campaign.

**What are the implications for public health practice?**

Given current recommendations in favor of mOPV2 use for VDPV2 response activities, countries should weigh the potential benefits of using fIPV against the operational challenges associated with its use. If countries determine that fIPV use is warranted, meticulous planning and preparation should precede such activities to ensure judicious use of the limited global stock of IPV.

## Footnotes

* http://www.who.int/workforcealliance/knowledge/case_studies/CS_Pakistan_web_en.pdf?ua=1.

† The World Health Organization’s open vial policy allows for the use of inactivated poliovirus vaccine (IPV) for up to 28 days after opening so long as the vaccine vial monitor is valid and the manufacturer’s instructions stipulate that it can be used as such. The vaccine vial must be within its expiry date, there should be no evidence of leakage or contamination, and it must be stored under proper conditions between vaccination sessions.
